# Molecular mechanism and physiological role of active–deactive transition of mitochondrial complex I

**DOI:** 10.1042/BST20130088

**Published:** 2013-09-23

**Authors:** Marion Babot, Alexander Galkin

**Affiliations:** *Queen's University Belfast, School of Biological Sciences, Medical Biology Centre, 97 Lisburn Road, Belfast BT9 7BL, U.K.

**Keywords:** active–deactive transition (A/D transition), mitochondrial complex I, hypoxia, ischaemia, thiol modification, thiol nitrosation, A/D transition, active–deactive transition, ROS, reactive oxygen species, SMP, submitochondrial particle(s)

## Abstract

The unique feature of mitochondrial complex I is the so-called A/D transition (active–deactive transition). The A-form catalyses rapid oxidation of NADH by ubiquinone (*k* ~10^4^ min^−1^) and spontaneously converts into the D-form if the enzyme is idle at physiological temperatures. Such deactivation occurs *in vitro* in the absence of substrates or *in vivo* during ischaemia, when the ubiquinone pool is reduced. The D-form can undergo reactivation given both NADH and ubiquinone availability during slow (*k* ~1–10 min^−1^) catalytic turnover(s). We examined known conformational differences between the two forms and suggested a mechanism exerting A/D transition of the enzyme. In addition, we discuss the physiological role of maintaining the enzyme in the D-form during the ischaemic period. Accumulation of the D-form of the enzyme would prevent reverse electron transfer from ubiquinol to FMN which could lead to superoxide anion generation. Deactivation would also decrease the initial burst of respiration after oxygen reintroduction. Therefore the A/D transition could be an intrinsic protective mechanism for lessening oxidative damage during the early phase of reoxygenation. Exposure of Cys^39^ of mitochondrially encoded subunit ND3 makes the D-form susceptible for modification by reactive oxygen species and nitric oxide metabolites which arrests the reactivation of the D-form and inhibits the enzyme. The nature of thiol modification defines deactivation reversibility, the reactivation timescale, the status of mitochondrial bioenergetics and therefore the degree of recovery of the ischaemic tissues after reoxygenation.

## Introduction

Production of energy in most aerobic cells is provided by the combined action of the mitochondrial respiratory chain and ATP synthase. Reducing equivalents from pyridine nucleotides (NADH) generated in several catabolic pathways are routed into the energy-converting respiratory chain via so-called complex I or NADH:ubiquinone oxidoreductase. This enzyme catalyses the oxidation of matrix NADH by membrane ubiquinone and is the major entry point for electrons into the respiratory chain. Unlike the 14-subunit prokaryotic enzyme, mitochondrial complex I contains approximately 30 additional so-called accessory subunits, many with still unknown functions. All known redox centres of this enzyme (FMN and FeS clusters) are located within core subunits in the hydrophilic domain of the enzyme, whereas proton translocation is carried out by several Na^+^/H^+^ antiporter-like subunits. Recent progress in structural studies [1,2] has led to significant improvements in the understanding of the coupling mechanism of this enzyme [1,3], whereas many aspects of regulation are still not completely understood. Dysfunction of complex I was found to correlate with a number of clinical conditions such as Leber's optic neuropathy, neuromuscular disorders, Parkinson's disease and the process of aging.

The unique property of the mitochondrial enzyme from several vertebrate species is the so-called A/D transition (active–deactive transition) [4,5]. In mammals, the catalytically competent A-form of the enzyme operates at physiological temperatures (>30°C) when substrates are available (*k* ~10^4^ min^−1^). When the enzyme is idle, it spontaneously converts into the dormant D-form. This form can potentially undergo reactivation given the availability of both substrates (NADH and ubiquinone). As a result of slow (*k* ~1–10 min^−1^) catalytic turnover(s), the D-form is converted back into the A-form and this process can be significantly slowed by the presence of fatty acids (in conjunction with Ca^2+^) and by alkaline pH [6,7].

## Structural differences between A- and D-forms of complex I

**Figure 1:**
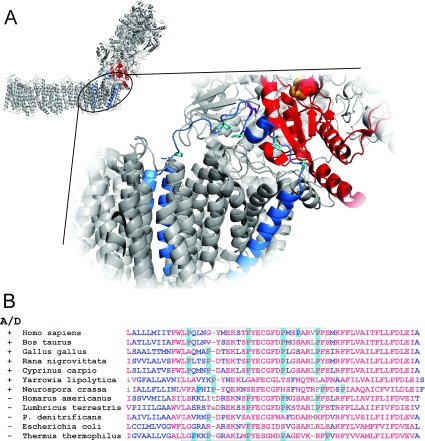
Location of the hydrophilic loop of Nqo7 subunit (*T. thermophilus* [1]), shown in blue, which is homologous with mitochondrially encoded subunit ND3 (**A**) The Ser^36^ homologue of Cys^39^ of ND3 is shown in purple and proline residues are shown in cyan. Note that this loop is located in close proximity to the quinone-binding site and terminal cluster N2. (**B**) Partial alignment of the homologues of ND3. Hydrophilic loop containing the critical Cys^39^ is shown in a box.

In spite of observed heterogeneity of mitochondrial complex I in the pioneering work of Estabrook and co-workers [8], very little is known about structural differences between the A- and the D-form of the enzyme. It was first established that upon thermal treatment (37°C), the NADH oxidase activity of the enzyme in SMP (submitochondrial particles) could be inhibited by thiol-group-specific reagents and this sensitivity was eliminated by incubation with substrates [9]. Later, the existence of the A- and the D-form was postulated for the enzyme in SMP and preparation of isolated complex I [10]. It is important to note that no differences in the polypeptide composition between the A- and the D-form of enzyme was later found [11,12], indicating that the sensitivity of the D-form is associated with the exposure of the cysteine-containing domain of known complex I subunit(s). To identify this subunit, Gavrikova and Vinogradov [11] used *N*-fluorescein maleimide to label the cysteine residue(s) that becomes exposed after deactivation of the enzyme in SMP. The conformation-specific labelling revealed a strong increase in fluorescence intensity in the region of 15 kDa only in the D-form. Using membrane-permeant [NEM (*N*-methylmaleimide)] and -impermeant [5,5′-dithiobis-(2-nitrobenzoic acid)] reagents, it was found that the critical cysteine residue accessible for chemical modification in the D-form was located at the matrix side of the enzyme [13]. This thiol group has subsequently been identified as Cys^39^ of the mitochondrially encoded subunit ND3 (Nqo7/NuoA) and is accessible only in the D-form of complex I [12]. This residue is indeed oriented towards the matrix and located in the hydrophilic loop connecting the second and third transmembrane segments of the ND3 subunit [12] (Figure 1A). Moreover, several pathogenic mutations close to Cys^39^ were found, indicating the importance of this region for complex I function [14–16].

Cys^39^ of ND3 is highly conserved among eukaryotes (conserved to 99% over 108 sequences) (Figure 1B). The corresponding prokaryotic Nqo7 subunit from *Paracoccus denitrificans* and NqoA from *Rhodobacter capsulatus* contains a homologue of the cysteine residue, whereas in *Escherichia coli* and *Thermus thermophilus*, it is replaced by serine. Composed of more than 40 subunits, the eukaryotic enzyme in vertebrates, such as cow [10], rat [17], mouse [18], chicken, frog and carp [5] displays the A/D transition as well as the 37–39-subunit complex I from fungi [5]. Despite the occurrence of the cysteine residue, the bacterial complex I from *P. denitrificans* and enzyme from non-vertebrate organisms, such as earthworm (*Lumbricus terrestris*), lobster (*Homarus americanus*) and cricket (*Acheta domesticus*), does not manifest patterns of the A/D transition [5]. Therefore the presence of this unique cysteine residue in the ND3 sequence does not correlate with the apparent ability of complex I to undergo the A/D transition (Figure 1B).

The fact that the A/D transition has a high activation energy [10] favours the idea of significant conformational changes upon deactivation. It has been reported that at least one other subunit might be involved in the A/D transition process. Indeed, the disruption of the 29.9 kDa subunit in *Neurospora crassa* (B13 in the bovine enzyme) led to a lower rate of thermal deactivation of complex I [19]. This hydrophilic subunit is well conserved from mammals to plants and fungi, but the function of B13 remains unknown.

Recently, using a 6.8 Å (1 Å=0.1 nm) heterobifunctional cross-linker, SPDP (succinimidyl 3-(2-pyridyldithio)propionate], we demonstrated that ND3 was closely located to the 39 kDa subunit (NDUFA9) in the D-form of complex I in SMP [20]. These two subunits formed a cross-linked product only in the D-form of the enzyme, and not in the A-form. This finding indicates that, upon deactivation, the position of Cys^39^ changes and it can be cross-linked to one of the lysine residues of the 39 kDa subunit in the vicinity. The latter is one of the accessory subunits from the family of heterogeneous short-chain dehydrogenase/reductases and contains a non-covalently bound NADPH molecule [21,22]. Therefore the 39 kDa subunit can be positioned at the interface between the membrane part and the hydrophilic part of complex I very close to the region of enzyme which is involved into A/D transition. This corresponds to the location of that subunit suggested for the isolated bovine enzyme [23,24]. Deletion of this subunit in yeast significantly destabilizes the complex I structure [21].

A close physical proximity of ND3 with 49 kDa (Nqo4) and PSST (Nqo6) subunits in *P. denitrificans* has been proved biochemically [25] and confirmed after resolution of the structure of the entire complex I from *T. thermophilus* [1]. Subunits PSST and 49 kDa are involved in the formation of terminal FeS cluster N2 and, along with ND3 and ND1, form a sealed quinone-binding cavity away from the membrane bilayer. The first transmembrane segment of ND3 is located in between two transmembrane segments of ND1 (Nqo8/NuoH) (Figure 1A). In the *T. thermophilus* enzyme, the hydrophilic loop containing Ser^46^ of Nqo7 (Cys^39^ in ND3) is part of the seal for the quinone-binding cavity [1]. Thus the hydrophilic interhelical loop of ND3 is a part of a crucial region where the energy of the redox reaction is transduced into conformationally driven proton translocation across the membrane part of the enzyme, probably via antiporter-like subunits [1]. The relocation of the hydrophilic part of ND3 upon deactivation of the mammalian enzyme could lead to a change in the quinone chamber, affecting interaction of the quinone headgroup with its binding site [1]. Partial opening of the well-sealed electron-transport pathway within the enzyme may explain the increased rate of ROS (reactive oxygen species) formation by the D-form of the enzyme [18,26].

The exact molecular mechanism exerting the A/D transition is unknown. One possibility is that a conformational change resulting in exposure of the critical Cys^39^ of ND3 involves other accessory subunits of the eukaryotic enzyme in the vicinity, e.g. 39 kDa subunit or B13 [19,20]. Alternatively, movement of the loop itself exerts the A/D conformational changes of complex I. In the bovine ND3 subunit, this loop is composed of 27 amino acids. Together with two proline residues that are located at the interface between the transmembrane helices, two to three other proline residues are found to be well-conserved in vertebrates (Figure 1B). Peptidylprolyl *cis*–*trans* isomerization has emerged as a conformational switch regulating processes such as ion channel gating [27] and protein domain movement ([28], and see [29] for review). It would be tempting to speculate that local bond rearrangement at the ‘hinge’ point could propagate through the protein backbone and would result in opening/closing of the flap formed by the loop. The isomerization is a slow reaction with an activation energy of approximately 100 kJ/mol occurring within the minute timescale [29] which corresponds to known parameters of A/D transition [10]. Introduction of negative charge as a result of deprotonation of the functional group close to the critical isomerizing proline residue significantly decelerates rotation of the imide bond and could explain the decrease in activation rate of complex I at higher pH [7]. Therefore the A/D transition would be regarded as an equilibrium between *cis* and *trans* isoforms of critical proline(s) in the hydrophilic loop of ND3. The energetically more stable *trans* prolyl bond could be adopted in the D-form, whereas isomerization results in closing of the flap and sealing of the quinone-binding cavity. On the other hand, conformational change induced by *trans*/*cis* proline isomerization in the hydrophilic loop affecting the position of the membrane helix or helices of ND3 cannot be excluded. The presence in the mitochondrial matrix of specific peptidylprolyl isomerases such as cyclophilin D, suggested to be associated with complex I [30], could be an important factor modulating the kinetics of the A/D transition *in situ*.

## Physiological effect of the A/D transition

**Figure 2:**
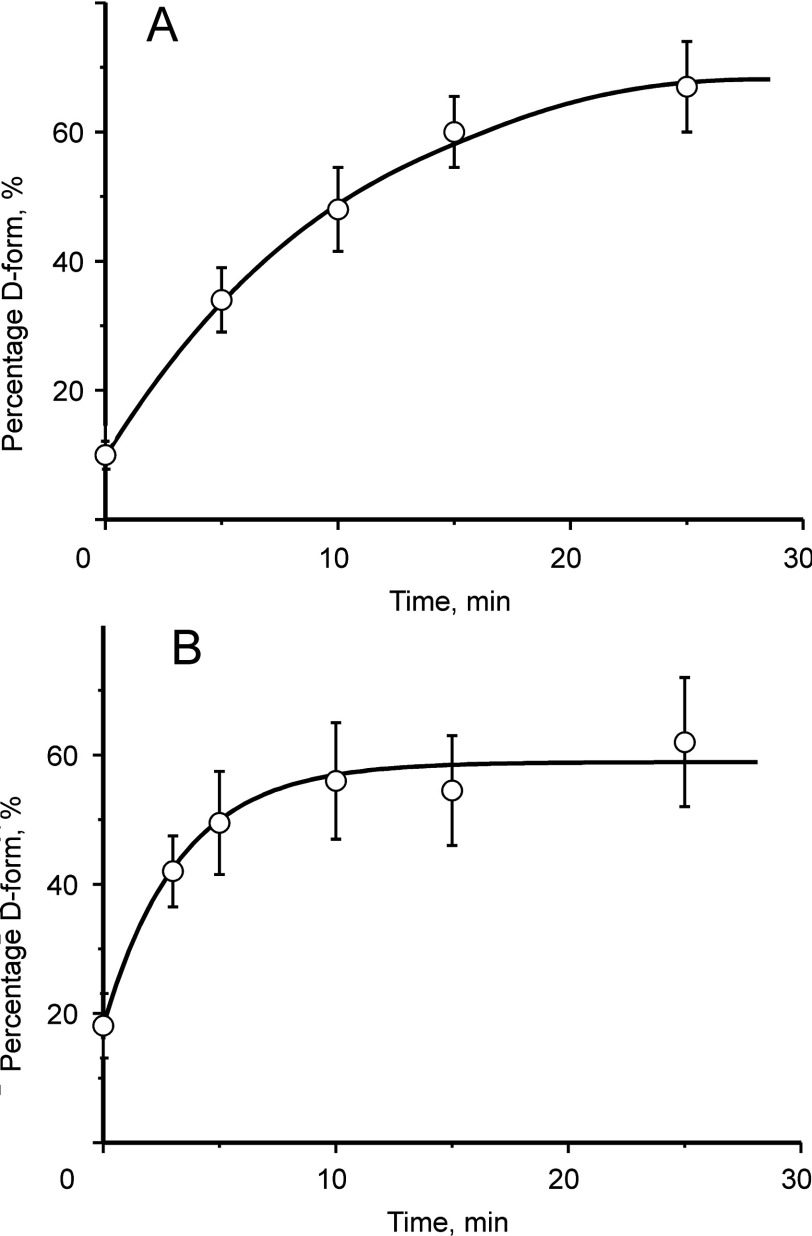
Time-course of de-activation of mitochondrial complex I in rat heart (**A**) and brain (**B**) tissue after cardiac arrest Percentage of the D-form in samples was determined as described in [18].

The physiological role of the A/D transition is still under discussion. Under conditions when the respiratory rate is decreased, e.g. at limited O_2_ concentration (or metabolic hypoxia [31]), the respiratory chain is reduced due to the slowing down of cytochrome *c* oxidase. As a result, the turnover of complex I becomes limited because of the lack of the electron acceptor ubiquinone. In this situation, the steady-state equilibrium in the A↔D reaction would be shifted to the right, and complex I in highly metabolizing tissues such as heart or brain would be readily converted into the D-form in a minute timescale [18,32] (Figure 2). In the Langendorf rat heart model and mouse infarction model, reperfusion results in the return of complex I A/D equilibrium to its initial level, indicating that reintroduction of oxygen causes reactivation of the D-form of the enzyme [18,32].

Therefore the A/D transition is a natural adaptive mechanism mediating mitochondrial response to hypoxia. In this section, we discuss several views on the role of the A/D transition of complex I.

(i) Initially, deactivation was considered as special mechanism preventing proton leakage to the mitochondrial matrix from the intermembrane space through the idle enzyme and decrease in protonmotive force [33]. However, later, it was found that the D-form of the enzyme in submitochondrial particles is still able to translocate protons during NADH oxidation by hydrophilic short-chain quinone analogues [34].

(ii) Recently, potential Na^+^/H^+^ antiporter activity was elegantly demonstrated for the D-form of bovine complex I in a reconstituted system and SMP [35]. There is a significant rise in intracellular Na^+^ concentration in cytoplasm upon oxygen deprivation [36]. It was suggested that the Na^+^/H^+^ exchange catalysed by the D-form accumulated during the ischaemic period and can contribute to the protection of mitochondrial ion balance [35]. However, on the basis of the redox-dependent proton-pumping mechanism proposed for complex I [1,3], the Na^+^ movement through the enzyme antiporter-like subunits is likely to operate via a different route for which the details are still not known. Moreover, it was demonstrated that increased Na^+^/H^+^ exchange in ischaemia leads to mitochondrial damage [37] and inhibition of antiporter activity protects mitochondria during reperfusion or attenuates oxidative stress in cardiomyocytes [38,39]. Decreased rates of overall Na^+^/Ca^2+^ exchange shown in mitochondria from ischaemic heart samples also suggest a decrease and not an increase in Na^+^/H^+^ exchange across the inner membrane when oxygen is lacking [40]. The role of mitochondria in regulating Na^+^ concentration in cytosol in ischaemia is not completely understood at present and more research is needed to elucidate the involvement of complex I in Na^+^ balance during ischaemia.

(iii) In ischaemia/reperfusion models, diminution of respiratory activity during reoxygenation would protect mitochondria from irreversible damage [41,42]. Therefore it is possible that slow activation of the D-form upon tissue reoxygenation may function as a protective valve and reduce the burst of respiration and consequent ROS production at the level of complex I or downstream sites. In addition, deactivation of the enzyme could prevent superoxide anion generation from the reduced flavin since the D-form does not catalyse reverse electron transfer from ubiquinol to FMN [7].

(iv) As shown previously, in tissues *ex vivo* [18,32] (but not in cells [43]), a significant fraction of the enzyme (5–15%) was found to be in the D-form at the physiological O_2_ concentration (Figure 2). Therefore, *in situ*, part of the energy released during steady-state NADH oxidation is used to maintain the catalytically competent A-state of the enzyme as suggested previously [4]. Maintenance of the fraction of complex I in the D-form would allow fast responses to changes in conditions such as a reductive pressure, ATP demand and oxygen availability by analogy with the well-known phenomenon of excess capacity of cytochrome *c* oxidase [44–46]. Therefore the A/D transition could be one of the mechanisms for fine-tuning enzyme activity in different tissues [5,18,47,48]. The time course of complex I deactivation after cardiac arrest reveals a much higher A/D transition rate in brain compared with heart tissue (Figure 2) and might explain the greater vulnerability of complex brain functions to oxygen deprivation.

Exposure of Cys^39^ in the D-form [11,12,49] can be very important in various pathological scenarios. Sensitivity of the D-form of complex I to covalent modification led to the proposal of possible regulation of enzyme activity via thiol-reactive natural effectors such as the GSH–GSSG couple [4]. Although no sensitivity of the D-form of the enzyme to reduced or oxidized glutathione was found in *in vitro* experiments [13,48], only the D-form of complex I was susceptible to inhibition by nitric oxide metabolites [48,49] and ROS [18]. Oxidation of Cys^39^ of ND3 in the D-form by H_2_O_2_ results in irreversible inhibition of the enzyme [18]. It was found recently that S-nitrosation of Cys^39^ of ND3 by mitochondrially targeted nitrosothiol MitoSNO protects cardiac tissue during ischaemia/reperfusion in mice *in vivo* [49]. Modification of cysteine thiols *in situ* depends on particular biochemical conditions that an attacking group could encounter: redox environment and hydrophobicity surrounding the target thiol, pH, ion composition, nitric oxide/O_2_ ratio and activity of enzymes mediating action of ROS and nitric oxide metabolites [50]. These conditions could vary significantly during oxygen deprivation in tissues. The magnitude of such modification of complex I is not clear at present. Prolonged exposure of the D-form of the enzyme to low steady-state levels of endogenous thiol-reactive molecules such as H_2_O_2_, S-nitrosoglutathione or peroxynitrite would lead to modification of some fraction of the enzyme. S-nitrosation of the ND3 subunit is reversible, probably via reduction by glutathione and thioredoxin systems [49], but nitration or oxidation is irreversible at the timescale of the ischaemia/reperfusion process (tens of minutes). Depending on the nature of modification, the fraction of the modified enzyme would gradually increase over time. Taking into account the high degree of flux control of complex I over oxidative phosphorylation [51,52], elimination of even a small fraction of the enzyme may lead to a significant decrease in ATP production by mitochondria, although no apparent effect is observed on activity of the respiratory chain [53]. Such modification of the D-form would result in the emergence of a mitochondrial population with altered ATP-generating capacity/ionic balance. Depending on the timeframe of the process and the size of such a population, recovery of the ischaemic tissues after reperfusion could be significantly affected.

## Conclusion

At a structural level, the mechanism of A/D conformational changes of eukaryotic complex I remains unclear, as its driving force and the involvement of accessory subunits are still unknown. It is very likely that the 39 kDa subunit is also involved in conformational changes upon deactivation.

Our observations suggest that accumulation of the D-form of the enzyme takes place in highly metabolizing tissues during ischaemia. A/D transition can be considered a natural intrinsic mechanism providing diminution of activity of the respiratory chain during the initial phase of reoxygenation and would protect mitochondria from irreversible damage. Cys^39^ of the ND3 subunit, exposed in the D-form, is susceptible to covalent modification by ROS and nitric oxide metabolites. It is possible that the accumulated D-form can react with natural effectors in mitochondria or with pharmacological agents during periods of hypoxia or reoxygenation, modulating the process of reactivation of the enzyme and outcome of ischaemia/reperfusion.
